# Flywheel Training Periodization in Team Sports

**DOI:** 10.3389/fphys.2021.732802

**Published:** 2021-11-08

**Authors:** Marco Beato, Sergio Maroto-Izquierdo, José L. Hernández-Davó, Javier Raya-González

**Affiliations:** ^1^School of Health and Sports Sciences, University of Suffolk, Ipswich, United Kingdom; ^2^Institute of Health and Wellbeing, University of Suffolk, Ipswich, United Kingdom; ^3^Department of Health Sciences, European University Miguel de Cervantes, Valladolid, Spain; ^4^Faculty of Health Sciences, Universidad Isabel I, Burgos, Spain

**Keywords:** isoinertial, eccentric, strength, soccer, handball, basketball, football

## Introduction

Strength training has a key role for performance and injury prevention purposes in team sports (Suchomel et al., 2016; Beato et al., 2021). Resistance training using isotonic exercises is the most popular methodology, however, this training method is concentric dominant, while the eccentric phase is generally underloaded. Because of the importance of eccentric contractions, one of the most commonly used methods in team sports to stimulate such a contraction is flywheel exercise (Maroto-Izquierdo et al., 2017b; Suchomel et al., 2019a; Beato and Dello Iacono, 2020). By means of a flywheel-rotating device, this training method allows for significantly increased eccentric force demands compared to traditional resistance training (Tesch et al., 2017; Beato and Dello Iacono, 2020). Further, when performing flywheel training with high inertial loads and following some instructions (e.g., to delay the braking action to the last third of the eccentric phase), greater eccentric than concentric force production can be achieved, which is known as eccentric overload (Norrbrand et al., 2010; Martinez-Aranda and Fernandez-Gonzalo, 2017; Piqueras-Sanchiz et al., 2020). This overloaded eccentric action has been suggested to have a major impact on acute responses and chronic adaptations and to be a key characteristic of flywheel training (de Hoyo et al., 2015; Beato et al., 2020; de Keijzer et al., 2020).

Although the implementation of flywheel training in sports is supported by the scientific evidence (discussed in the following sections), limited information is currently available about its periodization. Therefore, the aim of this article is to provide methodological bases for the periodization in team sports to practitioners. This paper is structured into four sections: (1) Rationale and benefits of flywheel exercise; (2) Strength training periodization in team sports; (3) Flywheel training periodization in team sports; and (4) Limitations and future directions of flywheel training periodization.

## Rationale and Benefits of Flywheel Exercise

Over the last decade, flywheel training has widely shown its usefulness to promote muscular hypertrophy and strength gains (Maroto-Izquierdo et al., 2017b; Nuñez and Sáez de Villarreal, 2017), alongside improvements in actions related to sports performance such as sprinting, jumping and changes of direction (Beato et al., 2019a; McErlain-Naylor and Beato, 2021a; Raya-González et al., 2021c). In addition, flywheel training has shown promising results for both rehabilitation (Romero-Rodriguez et al., 2011) and injury prevention purposes (Askling et al., 2003; de Hoyo et al., 2015; Beato et al., 2021). While several of these benefits have been reported in untrained and recreationally trained populations (Tesch et al., 2017; Raya-González et al., 2021b), a substantial body of research has reported significant increases in trained athletes. Thus, significant increases in sprinting performance have been shown in soccer (Askling et al., 2003; Tous-Fajardo et al., 2016), handball (Maroto-Izquierdo et al., 2017a; Sabido et al., 2017; Madruga-Parera et al., 2020), and volleyball (Monajati et al., 2021) players. Similarly, improvements in vertical jumping and change of direction performance have also been reported in highly trained athletes from different sports, including soccer, handball, rugby, and volleyball (Tous-Fajardo et al., 2016; Maroto-Izquierdo et al., 2017b; Sabido et al., 2017). Instead, literature assessing the effects of flywheel training in female athletes is scarce, although promising results have been recently reported (Raya-González et al., 2021b). Finally, although still relatively understudied, flywheel exercises have been recently proposed as a viable strategy to stimulate post-activation performance enhancements (Beato et al., 2019b, 2020; Cuenca-Fernández et al., 2019).

## Strength Training Periodization in Team Sports

The logical and systematic sequencing of multiple training factors in an integrative fashion to optimize specific physiological and performance outcomes at predetermined time points is defined as periodization (Cunanan et al., 2018). In team sports, the training program should balance the global needs of the team (i.e., competitions and training sessions) with the individual health and performance demands of each player, which in turn makes an art out of periodization. Thus, to prepare any team-sport athlete for competition, a multitude of factors must be considered, such as technical and tactical specific skills, organization objectives, player interactions and competitive schedules (Gable, 2006). In this integrated system, the physical demands of sports imply that the development of sport-specific physical capacities has a key role in sports periodization.

Particularly in team sports, the athlete's strength qualities provide the physical attributes needed to execute specific movements and skills (Suchomel et al., 2016). The physical nature of each sport will determine the extent to which strength is needed and the type of strength qualities required (Haff and Nimphius, 2012). Both team performance and individual physical development can improve throughout the season with the support and inclusion of an appropriate strength training program (Madruga-Parera et al., 2020). Sport scientists and practitioners should seek for training methods and conditioning strategies which, depending on the competitive moment, enable them to individually develop the different regions of the force-velocity curve in sport-specific movements while ensuring health maintenance (i.e., injury prevention) (Suchomel et al., 2019b; Madruga-Parera et al., 2020; Beato et al., 2021; McErlain-Naylor and Beato, 2021b). Training periodization must consider two key aspects for its development. Firstly, the training load components, which will determine the specificity of stimuli (Brazil et al., 2020). Intensity, volume, training frequency, and training variation (e.g., exercise selection and training mode) provide transfer to the sport and a continual stress for adaptation in line with the specific aims of the program (Brearley and Bishop, 2019; Raya-González et al., 2021). Secondly, the competitive calendar (microcycles) and the season period (mesocycles, macrocycles) will define not only the strength quality to train and the proper amount of load for each training session but also the strength training program characteristics (Gable, 2006).

## Flywheel Training Periodization in Team Sports

Despite the importance of rational training periodization to optimize the effects of strength training programs being demonstrated (Williams et al., 2017), to date no comprehensive review has been developed for flywheel training periodization within team sports (Beato and Dello Iacono, 2020). The appropriate management of training strategies (e.g., phase potentiation, planned overreaching) and training variables (e.g., intensity, volume, exercises selection) are key points to optimize long-term adaptations while reducing detrimental effects of fatigue and injury risk (Fry and Kraemer, 1997; Martinez-Aranda and Fernandez-Gonzalo, 2017). In addition, the relationship between training dose and subsequent performance adaptations is key information for practitioners. In this line, a training frequency of two to three sessions per week seems effective to reach significant positive adaptations (Maroto-Izquierdo et al., 2017b; Núñez et al., 2018; Suarez-Arrones et al., 2018). Therefore, during pre-season or periods with a single competition per week, a training frequency of two weekly sessions would allow for greater chronic adaptations. The first flywheel training session (match day [MD]-4) should be focused on injury prevention and strength development involving multiset exercises with high inertial loads, while the second session (MD-2) may have a focus on power development using lower inertial loads and a lower overall volume (e.g., combination of sets and repetitions). An example of this type of load distribution can be found in Table 1A, which reports a pre-season weekly program for a professional handball team with one scheduled match. Table 1B reports an example of an in-season weekly program for a professional soccer team (one match per week), which is characterized by the subdivision of the team into two groups (i.e., starters and non-starters) based on the players' involvement during the previous match. On MD+2 practitioners may plan a flywheel training session for non-starters focused on injury prevention and strength development using relatively high-inertial load (e.g., >0.050 kg·m^2^) and volume (e.g., 3–4 sets of 6–8 reps)—it is worth noting that intensity and volume variables depend on the exercise used and players' strength level. Starter players instead should be mainly recovering (within 48 h from the previous match), therefore flywheel training has not been prescribed for this group. On MD-4 (72 h after the match), starters should be ready to perform an intense flywheel training session, while non-starters, who have performed this type of session the day before, may have a flywheel session with a focus on power development. Before the conclusion of this microcycle, starters may perform a further session with a focus on power training to have two flywheel training sessions per week; this type of session may be shorter than normal (since the match is scheduled 48 h later) and may require the implementation of a micro dose of flywheel training (low-volume high-intensity, e.g., 1–2 sets x 2–3 exercises). On the other hand, lower training frequencies (i.e., one session per week) have been also reported as effective to stimulate positive physical and performance adaptations (Sabido et al., 2017; Coratella et al., 2019; Raya-González et al., 2021a).

**Table 1A:** A pre-season weekly program for a professional handball team (one friendly match per week).

	**Day of the week**					
**MD+1**	**MD+2**	**MD-4**	**MD-3**	**MD-2**	**MD-1**	**MD**
Day off	*Afternoon* In-court training	*Morning*Gym training**FW training—** **Injury** **prevention/Strength** *Afternoon*In-court training	*Morning* In-court training *Afternoon* In-court training	*Morning*Gym training**FW training—** **Power** In-court training	Morning In-court training	*Afternoon*Friendly Match

*FW, flywheel; MD, match-day; Bold: Flywheel training*.

**Table 1B:** In-season weekly program for a professional soccer team (one match per week).

	**Day of the week**					
**MD+1**	**MD+2**	**MD-4**	**MD-3**	**MD-2**	**MD-1**	**MD**
Day off	*Recovery/Compensatory (Differentiating between S and NS)* Gym training **FW training—Injury prevention/Strength (NS)** Injury prevention (S) In-field training (NS and S)	*Strength*Gym training**FW training—** **Injury** **prevention/strength** **(S) and Power (NS)**In-field training	*Endurance* In-field training	*Speed*In-field training Gym trainingFW training—Power(micro dose)^*^	*Activation* In-field training	Match

*S, starters; NS, non-starters; FW, flywheel; MD, match-day; Bold: Flywheel training*.

* *Some players may perform low-volume high-intensity flywheel training (e.g., 1–2 sets x 2–3 exercises)*.

Congested fixtures periods are common scenarios in professional team sports, in which players need to compete twice a week with a limited amount of time available for training. This hinders the implementation of more than one flywheel training session per week (Wing, 2018). Therefore, practitioners should be encouraged to plan a single session (in such a scenario) focused on power training and, whether appropriate conditions are given, to implement an additional micro dose of flywheel training (e.g., 1–2 sets x 2–3 exercises, see Table 1C) on MD-2. Despite the lack of studies comparing flywheel periodization using different training frequencies, it may be suggested that two sessions a week should be recommended during the pre-season period, while a single session per week should be the minimum dosage in-season. Please, (see Tables 1A–C) for examples of microcycles in sports (pre-season with one match per week, in-season with one match per week, and in-season with two matches per week, respectively).

**Table 1C:** In-season weekly program for a professional basketball team (two matches per week).

	**Day of the week**					
**MD+1**	**MD-2**	**MD-1**	**MD**	**MD-2**	**MD-1**	**MD**
*Recovery*In-courtGym trainingInjury prevention	*Strength* Gym training **FW training—** **Power** In-court training	*Activation*In-court training	Match	*Recovery*In-courtGym trainingFW training—power(micro dose)^*^	*Activation* In-court training	Match

*FW, flywheel; MD, match-day; Bold: Flywheel training*.

* *Some players may perform low-volume high-intensity flywheel training (e.g., 1–2 sets x 2–3 exercises)*.

To get an adequate configuration of flywheel training programs and, consequently, to rationally periodize such programs, it is necessary to know the available evidence-based guidelines (Beato and Dello Iacono, 2020). Regarding volume, flywheel training programs using multiple sets (between 3 and 6) and repetitions (between 6 and 8) have improved team sports athletes' performance, facilitating progression of this component during flywheel training periodization. Regarding intensity, previous research has shown that lower inertial loads (i.e., 0.025–0.050 kg·m^2^) may be suitable to produce higher movement velocity and, thereafter, promote power gains (Martinez-Aranda and Fernandez-Gonzalo, 2017; Sabido et al., 2018; McErlain-Naylor and Beato, 2021b), while higher inertial loads (i.e., >0.050 kg·m^2^) may be more suitable to develop strength-related characteristics. However, the right combination of different inertial loads is necessary to optimize athletic performance (e.g., power and force) and for the implementation of successful muscle injury prevention programs during pre- and in-season periods (Beato and Dello Iacono, 2020; Beato et al., 2021; Raya-González et al., 2021). Despite this, no clear evidence about long-term training-induced effects and exercise intensity manipulation in the flywheel training field are available, so future studies are warranted on this aspect. Additional variables, such as rest interval between sets, should be considered since they may affect both acute responses and chronic adaptations to strength training. To date, only one study has evaluated the influence of rest intervals between sets on power decreases during flywheel training (Sabido et al., 2020). As a general guide, it seems that the appropriate rest interval is influenced by the inertial load used. Thus, lower inertial loads allow for the use of shorter rest intervals (e.g., <2 min), whereas higher inertial loads require longer rest periods (e.g., >2–3 min). Finally, exercise selection should be considered by practitioners to optimally design their training programs. Multi-joint exercises such as the flywheel squats, deadlifts, and lunges should be prioritized in training sessions seeking strength and power development (Maroto-Izquierdo et al., 2017b; Beato and Dello Iacono, 2020; Madruga-Parera et al., 2020), in particular because greater transfer from strength training to sports performance occurs, while less functional single-joint exercises such as the flywheel leg curl and flywheel hip extension (Askling et al., 2003; Piqueras-Sanchiz et al., 2020; Suarez-Arrones et al., 2020; Beato et al., 2021) may be preferentially used as injury prevention exercises.

The specific selection of the above-mentioned training variables is “only” one step in the flywheel training programming. The magnitude and frequency of variations in the training content define the periodization model used. In this regard, previous studies have used linear periodization models (i.e., maintaining training load components stable throughout the program) (Gual et al., 2016; Sabido et al., 2017; Núñez et al., 2018), but most of them have applied non-linear periodization models (Askling et al., 2003; de Hoyo et al., 2015; Gonzalo-Skok et al., 2017; Raya-González et al., 2021a). In this sense, variations of weekly frequency or training volume throughout the flywheel program are the main common strategies. Practitioners may decide to manipulate the aforementioned training program components but also to apply tapering strategies (i.e., progressive reduction of the sets and repetitions) during the last weeks of the training program to optimize its effects (Raya-González et al., 2021a). Additionally, due to the special characteristics of team sports, not only periodization throughout the entire program should be performed, but periodization in the microcycle itself, considering the different phases of the season and the specific characteristics of each sport (see Tables 1A–C).

## Limitations and Future Directions of Flywheel Training Periodization

The existing body of evidence of flywheel training periodization suffers from some limitations. Firstly, no well-designed studies have compared long-term effects of different flywheel training periodization programs, therefore future studies are needed to deepen how flywheel training periodization can enhance its benefits. Secondly, flywheel training periodization should be adapted based on athletes' experience. Since amateur participants potentially have different requirements regarding training dose compared to professional athletes. Furthermore, most of the studies enrolled male athletes, therefore further research involving female athletes is warranted. Finally, knowledge of low flywheel (i.e., micro dose) weekly training volume and frequency on sport-related performance is scarce, so more research is needed on this topic.

## Conclusions

This article has provided, for the first time, some information and practical indications about flywheel training periodization in team sports. This paper has recapped the rationale for the use of flywheel training in sports, it has analyzed the most recent evidence and summarized some of the characteristics of strength training periodization, it has discussed how to periodize flywheel training in pre-season, in season, and during a congested fixture period in three different sports (giving some examples of microcycles). Finally, it has outlined the current strength and limitations of the literature on this argument, which can address researchers to design future studies aiming to evaluate the effect of flywheel training periodization in team sports.

## Author Contributions

All authors listed have made a substantial, direct and intellectual contribution to the work, and approved it for publication.

## Conflict of Interest

MB declares to have received financial support for his research from a private company, Desmotec, producing flywheel devices in 2020. The remaining authors declare that the research was conducted in the absence of any commercial or financial relationships that could be construed as a potential conflict of interest.

## Publisher's Note

All claims expressed in this article are solely those of the authors and do not necessarily represent those of their affiliated organizations, or those of the publisher, the editors and the reviewers. Any product that may be evaluated in this article, or claim that may be made by its manufacturer, is not guaranteed or endorsed by the publisher.
